# Population genetic diversity of an odorous frog *Odorrana grahami* (Amphibia: Anura: Ranidae) in relation to conservation based on mitochondrial DNA

**DOI:** 10.1080/23802359.2018.1536455

**Published:** 2018-11-26

**Authors:** Gui-Ying Chen, Bin Wang, Jiong-Yu Liu, Jian-Ping Jiang, Ping Gao

**Affiliations:** a College of Life Science, Sichuan University, Chengdu, China;; b Chengdu Institute of Biology, Chinese Academy of Sciences, Chengdu, China;; c College of Life Science, Sichuan Normal University, Chengdu, China

**Keywords:** Genetic diversity, population structure, mitochondrial DNA, *Odorrana grahami*

## Abstract

The diskless-fingered odorous frog *Odorrana grahami* is widely distributed in the high-altitude mountains in the southwestern China and northern Indochina regions. In this study, a comparative analysis of the mitochondrial COI gene sequences was performed to examine the population genetic diversity of 76 individuals from 10 localities across the distributional range of the species. Haplotype diversity and nucleotide diversity were 0.605 and 0.00199, respectively, in the total population. An AMOVA indicated that 47.3% of the total variation originated from variation within individual populations and 52.7% came from variation between the 10 geographic populations. Tests of neutral evolution indicated that a recent expansion occurred in total population. The findings provide useful information for the conservation of this species.

## Introduction

The odorous frog *Odorrana grahami* is a member of the family Ranidae. It is commonly found in the high altitude (1150–3200 m) mountain streams in southeastern Asia, ranging from southwestern Sichuan, western Guizhou, and Yunnan provinces of China to northwestern Myanmar and northern Vietnam of Indochina (Fei et al. 2012; Frost 2018). In its distributional range west to the edge of Tibetan Plateau, huge mountains and deep valleys in the Hengduan Mountains and Yunnan-Guizhou Plateau has been widely proposed to impede gene flow and promote speciation, especially in frogs (e.g. Che et al. 2010; Zhang et al. 2010). Thus, *O. grahami* is an ideal model for exploring historical phylogeographic processes in southeastern Asia.

In recent decades, due to a variety of human-caused threats, such as habitat destructions, excessive captures, and pollutions, the number of *O. grahami* has declined dramatically (Fei et al. 2012). In view of the serious threats and their high sensitivity to environmental factors, this species was listed as the NT (Near Threatened) species in the Red List of China’s Vertebrates (Jiang et al. 2016). However, information especially on the population diversification of this species remains scarce (Fei et al. 2012), and it is obligatory for us to develop strategies to figure out the status of the species for protecting it as soon as possible. Accordingly, investigations on population diversification of the species would play a significant role in the conservation of its endemic germplasm resources and genetic diversity.

In light of its rapid rate of evolution and maternal inheritance (Sun et al. 2012), Mitochondrial DNA (mtDNA) marker has been increasingly applied for evaluating the levels of genetic divergence, detecting barriers for gene flow, and recognizing phylogeographic lineages (Weiss et al. 2011; Liu et al. 2015; Wang et al. 2017). In this study, the mitochondrial cytochrome oxidase subunit I (COI) gene was used to explore the population genetic diversity and diversification of *O. grahami* from southwestern China.

## Materials and methods

**Figure 1:**
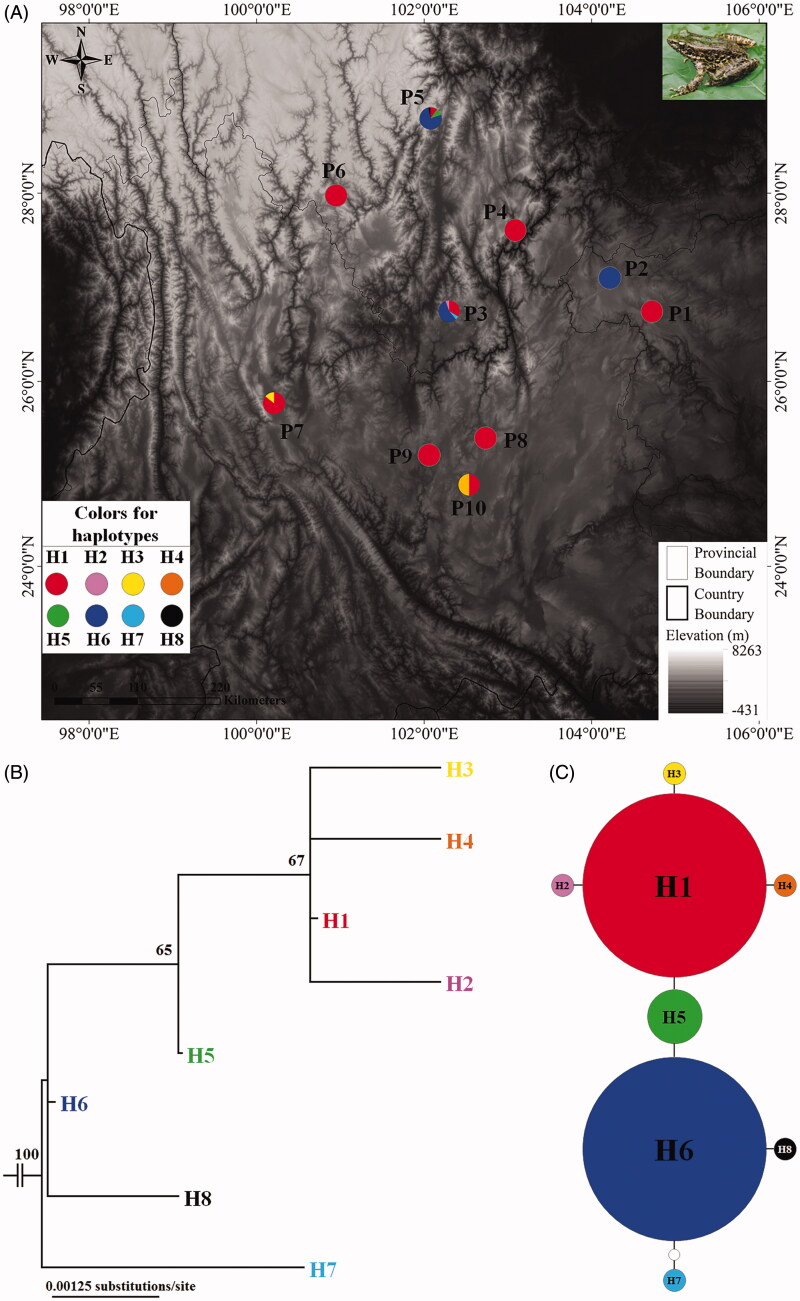
Sampling localities in this study and phylogenetic relationships of *Odorrana grahami*. (A) Sampling localities (P1–P10) in this study. Inset photo shows *O. grahami.* (B) Maximum likelihood tree based on COI gene sequences of haplotypes of *O. grahami*. Bootstrap supports with >50% values are denoted near nodes. (C) Haplotype network of *O. grahami*. The circle size is proportional to the number of samples. One white dot means one mutation.

A total of 76 specimens were collected from 10 localities (P1–P10) scattered in the distributional range of *O. grahami* in southwestern China (Figure 1(A); Table 1). All specimens were preserved in Chengdu Institute of Biology, Chinese Academy of Sciences. Tissues were collected and stored in 95% ethanol at −20 °C. Total genomic DNA was extracted from the tissues using a standard phenol–chloroform extraction procedure (Sambrook et al. 1989). Primers for amplifying COI gene was designed using the Primer-BLAST tool on NCBI web with default settings: forward primer 5′-TAGAWKGRCRGSCCTCGATCCCG-3′ and reverse primer 5′-GTRGTRTTAAGATTTCGGTC-3′. Amplifications were carried out in 50 μl reaction volumes containing 1 μl of genomic DNA, 27.5 μl of golden mix (Golden Easy PCR System; TaKaRa, Dalian, China), 1 μl of each primer, and 0.5 μl of Taq polymerase (TaKaRa, Dalian, China). PCR conditions were as follows: 95 °C for 3 min followed by 35 cycles of 95 °C for 30 s, 58 °C for 35 s, and 72 °C for 1 min, and 72 °C for 10 min as a final extension. All of the amplified products were purified with a Gel Extraction Kit (Generay Biotech (Shanghai) Co., Ltd., Shanghai, China) following the manufacturer’s instructions. The PCR products were sent to Shanghai Generay Biotech Co. Ltd. (Shanghai, China) for sequencing in both directions using the similar primers that were used in PCR.

**Table 1.** Genetic diversity and neutrality tests of *O. grahami* populations.

Population ID	Locality	Latitude	Longitude	*n*	*h*	*π*	Tajima’s *D*	Fu’s *Fs*
P1	Liupanshui City, Guizhou Province	26.6859°	104.6316°	3	0.000	0.00000		
P2	Weining County, Guizhou Province	26.8503°	104.2020°	1	0.000	0.00000		
P3	Huili County, Sichuan Province	26.7766°	102.2724°	18	0.608	0.00228	−0.24781	0.560
P4	Jinyang County, Sichuan Province	27.7258°	103.2743°	1	0.000	0.00000		
P5	Mianning County, Sichuan Province	28.9324°	102.2390°	30	0.405	0.00099	−0.53872	−0.858
P6	Yanyuan County, Sichuan Province	27.9936°	100.9901°	3	0.000	0.00000	−	−
P7	Dali City, Yunnan Province	25.8495°	100.0956°	7	0.286	0.00049	−1.00623	−0.095
P8	Kunming City, Yunnan Province	24.9658°	102.6075°	9	0.000	0.00000		
P9	Shuangbai County, Yunnan Province	24.2760°	101.7859°	2	0.000	0.00000		
P10	Yimen County, Yunnan Province	23.9649°	102.5524°	2	1.000	0.00170		
Total	–	–	–	76	0.605	0.00199	−0.71828	−1.788*

*n*: number of samples; *h*: haplotype diversity; *π*: nucleotide diversity.

Significance level: *p < .05.

Sequences were aligned using MAFFT 6 (Katoh et al. 2002) with default settings and manually checked. Variable sites, conserved sites, and nucleotide composition were estimated using MEGA 6 (Tamura et al. 2013). Haplotype diversity (*h*) and nucleotide diversity (*π*) were estimated using DnaSP 5 (Librado and Rozas 2009). Genetic signals of departure from neutrality or potential population expansion were estimated for populations using Tajima’s *D* (Tajima 1989) and Fu’ *Fs* (Fu 1997) statistics, estimated in DnaSP. Pairwise uncorrected *p*-distances between populations were estimated using MEGA. The hierarchical distribution of overall diversity was determined using an Analysis of Molecular Variation (AMOVA), as implemented in Arlequin 3.11 (Excoffier et al. 2005).

Phylogenetic relationships of haplotypes were reconstructed using maximum likelihood (ML), as implemented in the program PHYML 3.0 (Guindon et al. 2010). For the phylogenetic analyses, one COI sequence of *O. margaratae* was downloaded from GenBank (KJ815050) for use as outgroup. For ML analyses, the best-fitting nucleotide substitution model was selected by likelihood ratio tests under the Corrected Akaike Information Criterion (AICc) implemented in JMODELTEST 2.1.7 (Guindon and Gascuel 2003; Darriba et al. 2012). The optimal nucleotide substitution model (TrN + I + G) was selected for these analyses. Non-parametric bootstrapping with heuristic searches of 1000 replicates was used to assess confidences of branches in ML trees. In addition, haplotype networks were constructed using the maximum parsimony method in TCS 1.21 (Clement et al. 2000).

## Results

A total of 76 COI gene sequences were analyzed. Eight variable nucleotides were observed among 588 base-pair long sequence, thus accounting for a 1.36% proportion. The transition/transversion bias was 1.08, and the proportions of base types were A = 22.6%, T = 29.1%, C = 29.0%, and G = 19.3%, with (A + T) = 51.7% significantly higher than (C + G) = 48.3%. Eight haplotypes were recognized from 76 individuals of 10 populations of *O. grahami* (Table 1). All haplotype sequences were submitted to GenBank with accession numbers MH697562–MH697569. Haplotype H1 was shared by nine populations (except population P2), which occupied largest distribution range; haplotype H6 was shared by three populations (P2, P3 and P5); and other haplotypes were unique to each population (Figure 1(A); Table 2). Four haplotypes were observed in the P3 (H1, H2, H6 and H7) and P5 (H1, H5, H6 and H8), respectively; two haplotypes was observed in P7 (H1 and H3) and P10 (H1 and H4), respectively; and only one was found in each of other six populations (Table 2).

**Table 2.** Distribution and number of individuals of the eight haplotypes in ten population of *O. grahami*.

Population ID	Haplotype							
	H1	H2	H3	Η4	H5	H6	H7	H8
P1	3	–	–	–	–	–	–	–
P2	–	–	–	–	–	1	–	–
P3	6	1	–	–	–	10	1	–
P4	1	–	–	–	–	–	–	–
P5	3	–	–	–	3	23	–	1
P6	3	–	–	–	–	–	–	–
P7	6	–	1	–	–	–	–	–
P8	9	–	–	–	–	–	–	–
P9	2	–	–	–	–	–	–	–
P10	1	–	–	1	–	–	–	–

The estimated haplotype diversity and nucleotide diversity are shown in Table 1. The total haplotype diversity of the 10 populations was low with 0.605. The P10 population showed the highest haplotype diversity (1.0), followed by the P3 population (0.608), then P5 (0.405) and P7 populations (0.286), with the other six populations exhibiting the zero haplotype diversity because each of them occupied only one haplotype. The total nucleotide diversity of the 10 populations was low with 0.00199. The P3 population showed the highest nucleotide diversity (0.00228), followed by the P10 (0.00170), P5 (0.00099), and P7 (0.00049) populations, with the other populations exhibiting the zero nucleotide diversity.

In total population, Tajima’s *D* value was negative but not significant (*p* > .05), and Fu’s *Fs* value was significantly negative, suggesting the recent population expansion (Table 1). Although P5 and P7 presented negative Tajima’s *D* and Fu’s *Fs*, they were not significant. P3 showed negative Tajima’s *D* value and positive Fu’s *Fs* value with no significance (Table 1). The two indexes could not be estimated for other populations because insufficient polymorphisms in them.

The pairwise genetic distances between populations fall in the range of 0.000–0.425%, with the overall average at 0.22%. AMOVA showed that 52.7% of the molecular variance was attributed to the differentiation among populations, whereas 47.3% of the molecular variance was derived from within populations.

In the ML tree (Figure 1(B)), all samples of *O. grahami* were clustered into one clade. Haplotypes H1–H4 were clustered into one lineage (bootstrap supports, bs = 67), and then H5 was clustered with this lineage (bs = 65), but haplotypes H6–H8 were almost parallel at the base of the tree. Haplotype network presented: H1 and H6 were occupied by 34 samples, respectively; H1 was linked to H6 through H5; H2–H4 each independently linked to H1; and H7 and H8 each independently linked to H6 (Figure 1(C)).

## Discussion

This study explored the population genetic diversity of the mountain frog *O. grahami*, which was widely distributed from southwestern China to northern Indochina with a mass of enormous mountains and large deep valleys (Fei et al. 2012). This complicated geographical topology has been usually considered to supply significant barriers for gene flow and promote lineage diversification even for speciation (e.g. Zhang et al. 2010; Wang et al. 2017). Moreover, mitochondrial COI gene has been often used to examine population structure and often disclosed considerable divergence between lineages (Wang et al. 2017). However, our results based on the mitochondrial COI gene sequences revealed low genetic diversity in frog *O. grahami*. In this work, only eight haplotypes were recognized for 76 specimens from 10 populations across the range of *O. grahami*, and the haplotype H1 existed in all 10 populations except P2, indicating that all examined populations of the species might be derived from a common ancestor in recent evolution. Moreover, the genetic distance between these populations just reached average of 0.22% in a very low level. Further, AMOVA also suggested that approximately a half molecular variance was attributed to the differentiation within populations.

Of course, a certain level of divergence still existed in the species. Two haplotypes (H1 and H6) were indicated as the domain haplotypes in the populations. H1 was occupied by 34 individuals of nine populations and H6 was also in 34 individuals from three populations, and as noted, they were linked through the third haplotype (H5). This result indicated that the species has been possibly divided into at least two lineages. ML analyses also basically fitted this model. Merely, divergence of the lineages was still shallow (only two mutations; Figure 1(C)) or in the recent period.

Neutrality tests revealed that there was no signal for the recent population expansion in all populations, but there was a significant signal in the total population. The recent population expansion might be mainly derived from the expansion of haplotype H1, which existed in almost all populations. Of course, the recent expansion of H6 through part of populations could not be discounted. But, in the current situation, the ancestral region was still not uncertain because two geographical populations (P3 and P5) occupied at least three different haplotypes, which could be commonly indicated as the dispersal center (Figure 1(A)). Yet, we at least pointed out that the large region from the P3 to P5 localities had much probability to be the expansion center, and many populations especially the peripheral populations, such as P1and P6, had quite low genetic diversity (Figure 1(A); Table 1).

Totally, in *O. grahami*, the genetic diversity and diversification level were considerably low, indicating that it might be a young species. Population with low genetic diversity was usually proposed to be experienced bottleneck effects (Miracle and Campton 1995). Although the reasons of this kind of effects have not been disclosed for the species, we should pay much attention on its conservation. In the past decades, the population has been decreased mainly because of habitat destructions and excessive captures (Fei et al. 2009, 2012). Fortunately, in the past decades, in its distributional range, an increasing number of natural conservation areas has been drawn out, and the environment has taken a turn for the better. With the result of this study, we hope to supply useful genetic foundations for developing strategies on protecting *O. grahami* frogs and their habitats, for example, the populations P3 and P5 with higher genetic diversity should be key protected populations for this species.
